# Seasonal abundance, blood meal sources and insecticide susceptibility in major anopheline malaria vectors from southern Mauritania

**DOI:** 10.1186/s13071-018-2819-3

**Published:** 2018-04-10

**Authors:** Mohamed Aly Ould Lemrabott, Mohamed Salem Ould Ahmedou Salem, Khyarhoum Ould Brahim, Cecile Brengues, Marie Rossignol, Hervé Bogreau, Leonardo Basco, Driss Belghyti, Frédéric Simard, Ali Ould Mohamed Salem Boukhary

**Affiliations:** 1 Unité de Recherche Génomes et Milieux, Université de Nouakchott Al-Aasriya, Faculté des Sciences et Techniques, Nouveau Campus Universitaire, BP 5026 Nouakchott, Mauritania; 2 Laboratoire Biotechnologie et Environnement, Equipe de Parasitologie et Environnement, Département de Biologie, Faculté des Sciences, Université Ibn Tofail, BP 133 Kénitra, Morocco; 3 Maladies Infectieuses et Vecteurs: Ecologie, Génétique, Evolution et Contrôle (MIVEGEC), Institut de Recherche pour le Développement - Centre National de la Recherche Scientifique - Université de Montpellier, BP 64501, 34394 Montpellier, France; 4 Aix-Marseille Université, Institut de Recherche pour le Développement, Assistance Publique - Hôpitaux de Marseille, Service de Santé des Armées, Unité Mixte de Recherche Vecteurs - Infections Tropicales et Méditerranéennes (VITROME), Institut Hospitalo-Universitaire - Méditerranée Infection, 13005 Marseille, France; 5 Unité de Parasitologie et d’Entomologie, Institut de Recherche Biomédicale des Armées (IRBA), 13005 Marseille, France

**Keywords:** Malaria, Vector, Mosquitoes, *Anopheles* spp., Insecticide resistance, Transmission, Sahel, Mauritania

## Abstract

**Background:**

Malaria is endemic in the southernmost Sahelian zone of Mauritania where the major known mosquito vector is *Anopheles arabiensis*. Understanding seasonal population dynamics, feeding preferences and insecticide resistance status of these vectors in the area is essential to improve vector control measures implemented at a local scale. Here, malaria vector populations’ bionomics is described in two sentinel sites located in the Sahelian zone of Mauritania.

**Methods:**

Between September 2014 and December 2016, longitudinal entomological surveys were conducted in Kobeni (15°49'N, 09°24'W) and Rosso (16°30'N; 15°48'W), two localities in the southern Sahelian zone of Mauritania. Adult mosquitoes were collected using indoor pyrethrum spray catch (PSC). Morphological and PCR-based methods were used to identify the species, detect *Plasmodium* parasites and analyze blood meals in individual mosquitoes. WHO insecticide susceptibility tests were performed with malathion (5%), bendiocarb (0.1%), permethrin (0.75%) and deltamethrin (0.05%) using female *An. gambiae* (*s.l.*) reared from larval and pupal collections from natural breeding sites.

**Results:**

A total of 2702 *Anopheles* mosquitoes were collected by PSC during the study period comprising 2291 *Anopheles gambiae* (*s.l.*), 376 *Anopheles rufipes* and 35 *Anopheles pharoensis.* In Rosso, all mosquitoes from the *An. gambiae* (*s.l.*) complex were molecularly identified as *An. arabiensis* (*n* = 455/455, 100%). *Anopheles pharoensis* represented 2.5% (*n* = 35/1420) of the specimens collected by PSC in Rosso. In Kobeni, *An. arabiensis* was dominant (*n* = 278/301, 92.3%) and occurred together with *Anopheles coluzzii* (*n* = 18/301, 6%) and *An. gambiae* (*s.s.*) (*n* = 3/301, 1%). Two *An. coluzzii* × *An. arabiensis* hybrids were also detected (0.7%) in Kobeni, and *An. rufipes* was the only other *Anopheles* species found resting indoors (*n* = 376/1277, 29.4%). There was an average of 5.6 and 3.6 indoor resting female *An. gambiae* (*s.l.*) per room in Kobeni and Rosso, respectively. Indoor resting female *An. gambiae* (*s.l.*) mosquitoes in both sites fed most frequently on bovine blood (35.5% in Rosso and 37% in Kobeni). The proportion of *An. gambiae* (*s.l.*) mosquitoes that took human blood was significantly higher in Kobeni (HBI = 37%) than in Rosso (HBI = 5.6%) and 32% of *An. gambiae* (*s.l.*) mosquitoes contained blood from more than one host species. None of the 1414 tested mosquitoes in both sites were found positive for *Plasmodium* spp. sporozoites. WHO insecticide resistance tests revealed resistance to permethrin in the *An. arabiensis* population from Rosso (mortality = 64%) as well as reduced mortality to deltamethrin (mortality = 97%).

**Conclusion:**

This study provides updated information on the composition and dynamics of the malaria vector system in southern Mauritania where malaria is endemic. Such data are a necessary prerequisite to devise and implement tailored malaria elimination strategies in areas of low residual transmission.

## Background

Although the number of countries with ongoing malaria transmission has decreased from 106 in 2000 to 95 by the end of 2015, malaria remains the most important human vector-borne disease worldwide [1]. In Mauritania, malaria is endemic with unstable seasonal transmission and 181,000 presumed and confirmed cases reported in 2015 [2]. Malaria infections are mainly due to *Plasmodium falciparum* which prevails in the southern Sahelian zone [3–5], whereas *P. vivax* is most widespread in the Northern Saharan zone, including Nouakchott, the capital city [3, 6]. Studies conducted in Mauritania indicate that seventeen *Anopheles* species have been recorded throughout the country out of which four are considered as primary vectors and four as secondary vectors of malaria in Africa [7]. The primary vectors include *Anopheles arabiensis*, *An. gambiae*, *An. funestus* and *An. melas* whereas *An. pharoensis*, *An. coustani*, *An. ziemanni* and *An. wellcomei* are considered as secondary vectors [7]. Nevertheless, among the primary vectors, only *An. arabiensis* has been incriminated as a malaria vector in Mauritania with reported infection rates of 0.25 and 1.6% in the central region of Assaba and Nouakchott, respectively [8, 9].

Data on malaria vector bionomics such as species diversity, local spatial and seasonal distribution and dynamics, feeding preference, vector capacity and resistance to insecticides are of paramount importance in the epidemiology and control of malaria [10, 11]. Data on seasonal abundance of malaria vectors in a given area are a prerequisite for understanding their role in malaria transmission and for predicting disease outbreaks [12]. For instance, high densities of mosquito vector population have been shown to be associated with many malaria epidemics that occurred in Iran [13]. Moreover, identification of the source of blood meals of malaria vectors is critical to a better understanding of the degree of human-vector interaction (i.e. anthropophily) [14] and estimation of the capacity to transmit the disease. Resting behavior is also another parameter that determines whether mosquito population prefers to rest indoors (endophilic) or outdoors (exophilic) after blood-feeding. Both kinds of resting behavior were observed in *An. gambiae*, *An. arabiensis* and *An. funestus*, the major malaria vectors in sub-Saharan Africa, with considerable variation between and within species [15]. For instance, *An. gambiae* (*s.s.*) and *An. funestus* are thought to be largely endophilic and spend considerable time indoors [16, 17] although exophilic behavior has also been reported [18, 19]. In contrast, *An. arabiensis* is generally exophilic [19] although endophily has also been reported [16, 20]. Nonetheless, mosquito resting behavior is a very important variable for planning mosquito control, especially when insecticides are to be used in indoor residual spraying (IRS). Moreover, the rapid development of insecticide-resistant malaria vectors seriously threatens the progress made in malaria control to date due to the limited number of insecticide classes recommended by the World Health Organization (WHO) for use against adult mosquitoes in public health programs [21].

Data on malaria vector bionomics in Mauritania are scarce, and in most cases date back to more than 50 years ago [22, 23] although few entomological studies were recently conducted [8, 9]. The aims of the present study were to investigate the species composition, seasonal variation and feeding preference of *Anopheles* mosquitoes in the Sahelian zone in Mauritania and to assess their susceptibility/resistance status against insecticides available to the National Malaria Control Programme for their control.

## Methods

### Study sites

**Fig. 1:**
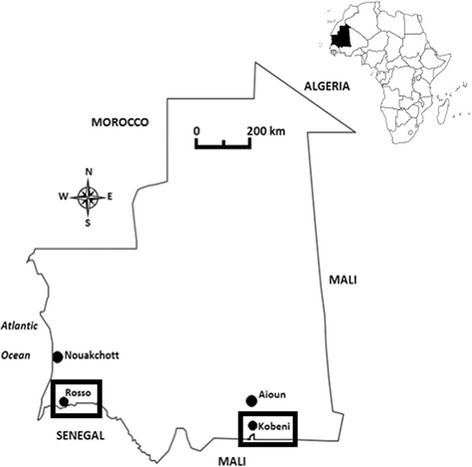
Map of Mauritania showing the study sites

Mosquitoes were sampled in the cities of Kobeni (15°49'N, 09°24'W, altitude 200 m above sea level) and Rosso (16°30'N, 15°48'W, altitude 8 m above sea level) in the provinces of Hodh Elgharbi and Trarza bordering Mali and Senegal, respectively (Fig. 1). Both localities belong to the Sahelian ecoclimatic zone characterized by a long dry season lasting from October to June and a short wet season extending from July to September. Rosso and Kobeni were selected for entomological studies because of their contrasting malaria epidemiological situation. Indeed, despite the presence of *Anopheles gambiae* (*s.l.*) as the main malaria vector and *P. falciparum* as the dominant causative parasite in the two study sites, malaria prevalence was shown to be low in Rosso [24], whereas a holoendemic malaria situation prevails in Kobeni [25].

Rosso, the major city of southwestern Mauritania and the regional capital of Trarza, is situated 204 km south of the capital city Nouakchott and has 57,000 inhabitants [26]. Its location along the Senegal River and the construction of numerous irrigation canals have contributed to the rapid propagation of irrigated agriculture, mainly consisting of rice cultivation. The city is bordered eastwards and westwards by large rice fields. Moors, Peulhs and Wolofs are the main ethnic groups of the city. The annual rainfall during the study period in Rosso was 77, 354 and 216 mm in 2014, 2015 and 2016, respectively. Mean relative humidity for the same period was 39, 65 and 62% and average temperature was 29 °C.

**Fig. 2:**
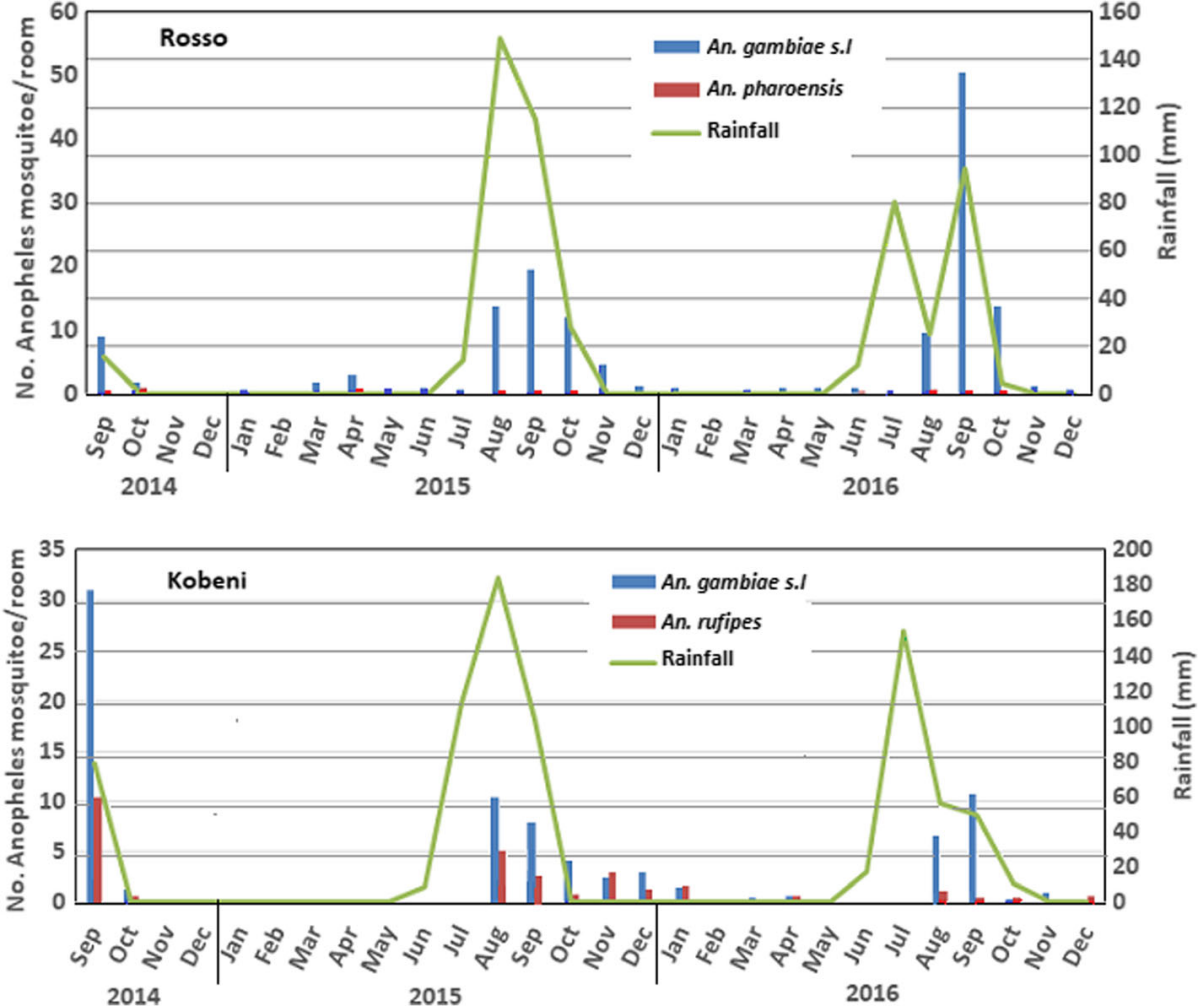
Monthly resting density of female *Anopheles* spp. and rainfall for the period 2014–2016 in two Sahelian sites, southern Mauritania

Kobeni, located around 20 km north of the Mali-Mauritania border, is the biggest district of the southeastern province of Hodh Elgharbi with 97,000 inhabitants according to the latest available population census data from 2013. During the rainy season of 2014, 2015 and 2016 the total rainfalls in Kobeni were 333, 409 and 250 mm, respectively. Data on the monthly rainfall during the study period were obtained from the national meteorological office (Fig. 2). Mean relative humidity throughout the study period was 47.5% (ranging from 19% in May-June to 76% in August-September) and the average temperature was 35 °C (ranging from 28 °C in January-February to 44 °C in April-May) [5]. Major human activities in Kobeni are livestock rearing (mainly cattle, sheep and goats) and rain-fed agriculture (mainly millet, sorghum and bean). A semi-permanent large pond is located near the city. Moors, Pulars, Soninkes and Bambara are living in Kobeni.

Dwellings in Kobeni and Rosso include traditional huts with mud walls and thatched roofs and modern buildings with cement walls and corrugated iron roofs. Domestic animals in both study sites are generally kept in fences close to human habitations. There are no precise data on the relative abundance of each domestic animal species but cattle and small ruminants (goats and sheep) are more abundant than humans in Kobeni in comparison with Rosso.

### Mosquito sampling and field processing

Indoor resting mosquitoes were collected by pyrethroid spray catches (PSC) [27]. PSCs were carried out for 28 months (September 2014 - December 2016) in seven randomly selected rooms per site, twice per month during the wet season (July-September) and monthly during the dry season (October-June). Mosquitoes were first visually sorted and grouped by genus (*Anopheles*, *Culex* or *Aedes*) and sex. Only female *Anopheles* specimens were then identified to species using standard taxonomic keys [28, 29]. Their gonotrophic status was determined as unfed, blood-fed, half-gravid or gravid. Specimens were stored individually in numbered microtubes containing silica gel and kept at -20 °C until further processed in the laboratory in Nouakchott.

### DNA isolation and species identification

Genomic DNA was isolated from legs and wings of individual *An. gambiae* (*s.l.*) mosquitoes using 2% cetyltrimethylammonium bromide (CTAB) solution [20 mM Tris-HCl pH 8; 10 mM ethylenediamine tetraacetic acid (EDTA); 1.4 mM NaCl; 2% N-acetyl-N,N,N,-trimethyl ammonium bromide] according to the method described by Delatte et al. [30]. *Anopheles gambiae* sibling species were identified using the multiplex PCR protocol developed by Scott et al. [31]. *Anopheles gambiae* and *An. coluzzii*, formerly known as S and M forms of *An. gambiae*, respectively, were differentiated using the short interspersed elements (SINE 200) approach, according to Santolamazza et al. [32].

### Blood meal source identification

The origin of the blood meal was assessed in fully blood-fed female mosquitoes using the protocol developed by Kent & Norris [33]. This multiplex PCR allows detection of human, bovine, goat, sheep, donkey and dog blood based on the amplification of a segment of the cytochrome oxydase *b* (*cytb*) gene, which varies in size.

### Insecticide susceptibility test

Mosquito immature stages were collected in Rosso and Kobeni in September-October 2015 and 2016 corresponding to the rainy season, through dipping from natural breeding sites consisting of highly productive rainwater puddles. Once collected, the larvae and pupae were transported in plastic bottles to the laboratory where they were reared at 26 ± 2 °C and 70 ± 5% relative humidity. Pupae were transferred into plastic cups and placed in cages for emergence of adult mosquitoes. Emerged adults were provided with sterile 10% sugar solution and morphologically identified using a taxonomic key [29]. Test papers impregnated with four WHO-certified insecticides, namely permethrin (0.75%), deltamethrin (0.05%), bendiocarb (0.1%), and malathion (2%), were used to perform the standard WHO adult bioassay [34]. Two-day-old female *An. gambiae* (*s.l.*) mosquitoes were exposed to the insecticide-impregnated or to the control oil-impregnated papers per batch of 20–25 sugar-fed female adults (i.e. four treatment and two control replicates per test). Observation of the number of knocked-down mosquitoes was made during one hour-long exposure period at regular intervals, after 10, 20, 30, 40, 50 and 60 min. Mosquitoes were then transferred into recovery tubes and kept under laboratory conditions with access to water and 10% sugar solution. Total mortality was recorded after 24 h. When mortality in the control group was between 5–20%, Abbott’s formula [35] was used to correct mortality estimates in test groups.

### Molecular detection of *Plasmodium* spp. in resting mosquitoes

DNA was extracted from dissected head and thorax of 1414 female mosquitoes and screened for *P. falciparum*, *P. vivax*, *P. malariae* and *P. ovale* DNA using the quantitative PCR assay with EvaGreen dye described in Mangold et al. [36]. Dissociation curves were used to estimate the specific melting temperature for each reaction. PCR amplifications were conducted in a LightCycler® 480 (Roche Applied Science, Mannheim, Germany).

### Data analysis

Indoor resting densities (IRD) were calculated for each species and locality as the arithmetic mean of the number of resting female mosquitoes collected per sprayed room in that locality at a given sampling date.

The human blood index (HBI) of engorged mosquito was estimated as the proportion of freshly fed mosquitoes found to contain human blood [37]. Mixed blood meals that contain human blood were included in the HBI calculation.

The fed to gravid ratio (F/G ratio) was calculated by dividing the total number of freshly fed mosquitoes by the total number of gravid and semi-gravid mosquitoes [27]. A F/G ratio > 1 indicates that females mosquitoes likely exit rapidly after a blood meal, suggesting exophilic behavior. In contrast, if the ratio is constantly less than 1, it indicates indoor resting tendency of the species (endophily).

Resistance/susceptibility of the tested mosquito populations was determined for each insecticide according to WHO criteria [34]. Mortality rates below 80% after 24 h observation period are indicative of resistance, whereas mortality rates above 98% indicate full susceptibility to the insecticide under scrutiny. When mortality rates range from 80 to 98%, resistance is suspected and should be further confirmed. Average IRD, F/G ratios, and HBI proportions were compared using z-statistics or t-statistics depending on the sample size. The 95% confidence intervals of the mortality rates of insecticide-exposed mosquitoes were calculated using Excel. A log-probit analysis was performed according to the method of Finney [38] using the R script [39], to compute KDT50 and KDT95 defined as the time taken to knock down 50 and 90% of mosquitoes, respectively. Statistical analyses of IRD, F/G ratios and HBI were performed using SPSS version 20 (SPSS Inc., Chicago, IL, USA) [40]. A probability value (*P*) of < 0.05 was considered statistically significant.

## Results

### Distribution and abundance of malaria vectors

A total of 2702 *Anopheles* mosquitoes (1277 in Kobeni and 1425 in Rosso) were collected by spray catch in the two selected study sites from September 2014 to December 2016 (Table 1). Morphological identification showed that the indoor resting mosquito fauna was essentially composed of *An. gambiae* (*s.l.*) mosquitoes in both sites, representing 97.8% of the total collections in Rosso and 70.6% in Kobeni. Specimens of *Anopheles pharoensis* (*n* = 35, 2.2%) were also found in Rosso. In Kobeni, a large proportion of *An. rufipes* (*n* = 376, 29.4%) was also found resting indoors whereas no specimen of *An. rufipes* was collected in Rosso (Table 1).

**Table 1:** Diversity of indoor resting female *Anopheles* spp. collected using pyrethrum space-spray catches between 2014 and 2016 from two Sahelian sites in Mauritania

		Rosso *n* (%)	Kobeni *n* (%)
*Anopheles* spp*.*	*An. gambiae* (*s.l.*)	1390 (97.5)	901 (70.6)
	*An. pharoensis*	35 (2.5)	0
	*An. rufipes*	0	376 (29.4)
	Total	1425 (100)	1277 (100)
*An. gambiae* (*s.l.*) *complex*	*An. arabiensis*	455 (100)	278 (92.3)
	*An. gambiae*	0	3 (1)
	*An. coluzzii*	0	18 (6)
	Hybrid^a^	0	2 (0.7)
	Total	455 (100)	301 (100)

^a^*An. arabiensis* + *An. coluzzii*

Molecular identification of *An. gambiae* sibling species was carried out in 455 and 301 randomly selected mosquito specimens from Rosso and Kobeni, respectively. In Rosso, only *An. arabiensis* was detected, while in Kobeni, *An. arabiensis* (278/301; 92.3%) occurred together with *An. coluzzii* (18/301; 6%) and *An. gambiae* (3/301; 1%). Two specimens (0.7%) were also identified as *An. arabiensis × An. coluzzii* hybrids in this locality (Table 1).

In Rosso, indoor resting *An. arabiensis* mosquitoes were captured throughout the study period (2014–2016) while in Kobeni, little or no specimens were captured indoor during the dry season (Fig. 2). Indoor resting densities of *An. gambiae* (*s.l.*) peaked during the rainy season with highest values observed in August and September in both sites during the study period, rising up to 50 (in September 2016) and 31 (in September 2014) specimens/room in Rosso and Kobeni, respectively. Peaks in mosquito abundance were observed concomitantly with rainfall peaks in Rosso, or with a slight delay in time in Kobeni. The overall indoor resting density of *An. gambiae* (*s.l.*) during the study period was significantly higher (*z* = 2.96, *P* = 0.003) in Rosso (IRD = 5.6 mosquitoes/room; *n* = 245 rooms) than in Kobeni (IRD = 3.6 mosquitoes/room; *n* = 245 rooms). *Anopheles pharoensis* was observed throughout the year in Rosso, although at low IRD (< 1.5 mosquitoes/room). In Kobeni, *Anopheles rufipes* occurred together with *An. gambiae* (*s.l.*) with IRD ranging from 0 during the late dry season (May-July) to 3 mosquitoes/room during the late rainy season/early dry season (August-January).

### Abdominal appearance and blood meal identification

Table 2 shows the results of abdominal inspections for all the indoor resting female mosquitoes collected in Kobeni and Rosso throughout the study. In Rosso, the fed to gravid (F/G) ratio of both *An. arabiensis* and *An. pharoensis* were below 1 suggesting mainly exophilic behavior. By contrast, in Kobeni, the F/G ratios of *An. gambiae* (*s.l.*), including mainly *An. arabiensis*, and *An. rufipes* were higher than 1, suggesting endophilic behavior of these malaria vectors in this locality. However, the F/G ratios of *An. gambia*e (*s.l.*) mosquitoes from Kobeni (F/G = 1.3) and Rosso (F/G = 0.76) were not statistically different (*t*_(17)_ = 1.83, *P* = 0.09).

**Table 2:** Abdominal status of indoor resting female *Anopheles* collected using the pyrethrum space-spray sheet method in two Sahelian sites in Mauritania

Study site	Abdominal status	*Anopheles* spp. *n* (%)		
		*An. gambiae* (*s.l.*)	*An. rufipes*	*An. pharoensis*
Rosso	Total	1390	–	35
	Unfed	249 (17.9)	–	11 (31.4)
	Blood-fed	495 (35.6)	–	4 (11.4)
	Gravid	117 (8.4)	–	10 (28.5)
	Semi-gravid	529 (38.1)	–	10 (28.5)
	F/G ratio	0.76		0.2
Kobeni	Total	901	376	–
	Unfed	96 (10.6)	50 (13.3)	–
	Blood-fed	456 (50.6)	207 (55)	–
	Gravid	110 (12.2)	22 (5.8)	–
	Half-gravid	239 (26.5)	97 (25.8)	–
	F/G ratio	1.3	1.7	

*Abbreviations*: F/G ratio, fed to gravid ratio

A total of 253 blood meals from resting female *An. arabiensis* collected in the two study sites (107 from Rosso and 146 from Kobeni) were tested by PCR to determine the source (Table 3). Resting female *An. arabiensis* in the two study sites had taken their blood meals from cattle (36%), donkey (14.6%), human (13%), goat (3.2%) and dog (0.8%). Overall, 81 (32%) *An. arabiensis* fed on multiple hosts among which 27 (33.3%) contained blood of human origin. HBI of *An. arabiensis* mosquitoes from Kobeni (HBI = 37%, *n* = 146) and Rosso (HBI = 5.6%, *n* = 107) were statistically different (z = 5.8, *P* < 0.001) and suggested low anthropophily.

**Table 3:** Source of blood meals of indoor resting female *An. arabiensis* in two Sahelian sites in Mauritania

Blood meal source	Study site		Total
	Rosso *n* (%)	Kobeni *n* (%)	
Single host	78 (72.9)	94 (64.4)	172 (68.0)
Human	4 (3.7)	29 (19.9)	33 (13.0)
Cattle	38 (35.3)	54 (37.0)	92 (36.3)
Donkey	31 (29.0)	6 (4.1)	37 (14.6)
Goat	3 (2.8)	5 (3.4)	8 (3.2)
Dog	2 (1.9)	0	2 (0.8)
Multiple hosts	29 (27.1)	52 (35.6)	81 (32.0)
Human × cattle	0	10 (6.8)	10 (3.9)
Human × donkey	0	1 (0.7)	1 (0.4)
Human × others	2 (1.9)	14 (9.6)	16 (6.3)
Other × other	27 (25.2)	27 (18.5)	54 (21.3)
Total	107 (100)	146 (100)	253 (100)
HBI^a^ (%)	5.6	37	23.7

^a^HBI is the number of blood meals containing human blood (single and multiple hosts) over the total number of blood meals analyzed

### Insecticide susceptibility

*An. gambiae* (*s.l.*) mosquitoes in Kobeni and Rosso were fully susceptible (100% mortality) to bendiocarb and malathion. They were also susceptible to deltamethrin (100% mortality) and permethrin (98.6% mortality) in Kobeni (Table 4). However, in Rosso, tested *An. arabiensis* mosquitoes were resistant to permethrin (64% mortality) and suspected to be resistant to deltamethrin (97% mortality).

**Table 4:** Mortality rates and knockdown times for *An. gambiae* (*s.l.*) populations exposed to different insecticides in two Sahelian sites in Mauritania

Insecticide	Rosso				Kobeni			
	n	% mortality (95% CI)^a^	KDT_50_ (95% CI)	KDT_95_ (95% CI)	n	% mortality (95% CI)^a^	KDT_50_ (95% CI)	KDT_95_ (95% CI)
Deltamethrin 0.05%	100	97 (95–99)	17 (15–18)	63 (54–77)	80	100	14 (5–20)	91 (52–580)
Permethrin 0.75%	100	64 (60–68)	31 (26–38)	592 (297–2007)	75	98.6 (96–100)	17 (14–20)	141 (98–248)
Malathion 5%	100	100			100	100		
Bendiocarb 0.1%	100	100			100	100		

^a^Mortality rate 24 hr post exposure to the insecticides with 95% confidence intervals in parentheses

*Abbreviations*: *n,* number of mosquitoes tested; KDT50 and KDT95, knockdown time in minutes for 50 and 95% mortality, respectively

### *Plasmodium* infection

None of the 1414 *Anopheles gambiae* (*s.l.*) tested for *P. vivax*, *P. falciparum*, *P. ovale* and *P. malariae* sporozoite infections was found positive.

## Discussion

The scarcity of data on malaria vector diversity, bionomics, insecticide susceptibility status and possible role in malaria transmission, particularly in the Sahelian malaria endemic zone, is a major hindrance for the implementation of an efficient malaria control programme in Mauritania. The present study documented the presence of *An. arabiensis*, *An. gambiae*, *An. coluzzii*, *An. rufipes* and *An. pharoensis* in the southern Sahelian zone of Mauritania. Within the *An. gambiae* complex, analyses also revealed the presence of two interspecific *An. coluzzii* × *An. arabiensis* hybrid females, accounting for 0.7% of the mosquitoes collected in Kobeni. Such frequency of hybrids is in agreement with the results of a recent meta-analysis of cross-species mating and hybridization within the *An. gambiae* complex in Africa [41]. Presence of these hybrids also highlights opportunities for gene flow between mosquito species within the *An. gambiae* complex, with possible introgression of insecticide resistance genes or other genetic traits of epidemiological or adaptive importance.

In Rosso, mosquitoes were present throughout the year in sampled dwellings, although with strong seasonal variation in abundance, whereas in Kobeni, no mosquito was collected during the driest and hottest months, at the end of the long dry season. The presence of *An. pharoensis*, a species known to breed in rice fields and generally associated to large swamps with vegetation, together with the year-round occurrence of *An. arabiensis* in Rosso, suggests permanent mosquito breeding is possible in the surrounding irrigation schemes or along the banks of River Senegal, as observed in other areas of Sahelian sub-Saharan Africa [42–46]. However, these putative breeding sites contribute little to the local vector production and the overall vector population dynamics appears to be driven by rainfall dynamics. Nonetheless, irrigated areas around Rosso might still represent a refuge where these vector populations can develop at a low rate during the harsh dry season, allowing rapid population expansion as soon as the rains start again for the next wet season [45, 46]. *Anopheles pharoensis* is a known vector of human *Plasmodium* in Africa [28, 29] and sporozoite-positive specimens were found in the Senegal River Delta [47]. Therefore, this mosquito might play a role as a secondary malaria vector in Rosso, although none of the specimens collected within the frame of our study was found infected with *Plasmodium*. Further studies are required to specifically investigate the epidemiological importance of this mosquito in residual malaria transmission in this area.

On the other hand, *An. rufipes*, which was collected in great numbers in Kobeni (i.e. 30% of the anopheline mosquitoes collected indoor in this locality) is known to be predominantly zoophilic, feeding mainly on cattle and non-human vertebrates, while often found resting inside human dwellings [28]. It is therefore unlikely to contribute to malaria transmission in the area.

Results also confirmed the wide distribution of *An. arabiensis* in the country [4, 5, 7–9]. However, despite the number of *An. arabiensis* mosquitoes analyzed using a highly sensitive quantitative PCR protocol [36], none of them were found positive for malaria parasites. Analyses of the blood meal sources revealed a very high proportion of non-human hosts and, consecutively, a very low human blood index for *An. arabiensis* in both Rosso and, to a lower extent, in Kobeni (HBI = 5.6 and 37% for *An. arabiensis* in Rosso and Kobeni, respectively). Although indoor resting mosquitoes were collected in the present study, our findings suggest that *An. arabiensis* exhibits both exophagic and endophagic behaviors. These results may have an important repercussion on vector control interventions, as exophagic vectors are more difficult to control by insecticide-impregnated bednets and indoor spraying of insecticides but are more amenable to control by interventions directed against the breeding sites. Many environmental factors, including host availability, host accessibility and innate host preference of mosquito, influence the final host selection of *An. arabiensis* and other members of the *An. gambiae* complex in Africa, which in turn modifies their blood-feeding behavior [48]. For instance, Sharp & Lesieur [49] demonstrated that *An. arabiensis* shifts from feeding on humans to feeding on livestock in response to indoor residual spraying of insecticides for vector control. Apparent zoophilic feeding behavior of *An. arabiensis* was already noticed in the area [8, 9].

Moreover, a low fed to gravid ratio as observed in Rosso indicates that a high proportion of females complete part of their gonotrophic cycles outdoors, suggesting exophilic behavior of the species. Our sampling design targeted only the endophilic fraction of the resident mosquito population in both sites, and it is therefore highly recommended to extend sampling to the outdoor biting/resting mosquito population in order to explore the possibility of residual malaria transmission occurring primarily outdoors. Indeed, previous studies showed natural sporozoite infections in *An. arabiensis* in the area, suggesting the vector likely contributes to malaria transmission [8, 9].

This study also revealed the presence of resistance to permethrin and suspected resistance to deltamethrin in *An. arabiensis* mosquitoes in Rosso and their full susceptibility to malathion and bendiocarb. However, in Kobeni, *An. arabiensis* mosquitoes were susceptible to all four insecticides tested. Insecticide susceptibility/resistance status of malaria vector in the Sahelian zone of Mauritania has never been assessed, except in one study that found full susceptibility (100% mortality rate) of *An. arabiensis* to permethrin in Rosso, Boghé, Sélibaby and Aioun and full susceptibility of *An. pharoensis* to deltamethrin in Rosso and Boghé [Traoré SF, unpublished WHO mission report, 2002]. The results presented in this study therefore suggest spread and selection for resistant mosquito population in Rosso, where the use of insecticide-based vector control methods together with the use of insecticide in agriculture, might be more intensive than in Kobeni (Ould Mohamed Salem Boukhary, personal communication). Since the current vector control measures are essentially based on the widespread distribution and use of pyrethroid-impregnated bed nets, regular surveillance of insecticide susceptibility of *An. arabiensis* is of utmost importance for an effective national malaria control programme.

## Conclusions

Sampling indoor resting mosquitoes in Rosso and Kobeni showed the predominance of *An. gambiae* (*s.l.*) (mostly or exclusively *An. arabiensis*), as well as smaller proportions of *An. pharoensis* (in Rosso) and *An. rufipes* (in Kobeni), and revealed highly zoophilic and putative exophilic preferences for the major malaria mosquito *An. arabiensis* in southern Mauritania where malaria is endemic and seasonal. Resistance to permethrin and, to a lesser extent deltamethrin, occurs in *An. arabiensis* captured in Rosso, but not in Kobeni. Further investigations on residual malaria transmission dynamics are required in this area for efficient disease and vector control strategies.

## Abbreviations

HBI: Human blood index
IRD: Indoor resting density
IRS: Indoor residual spraying
KDT: Knockdown time
PSC: Pyrethrum space-spray catch
WHO: World Health Organization
